# Complete genome sequences of mycobacteriophages JayJay and Rinkes

**DOI:** 10.1128/mra.01199-24

**Published:** 2025-01-15

**Authors:** Dale Liebenberg, Nday L. Sungu, Edith E. Machowski, Christopher S. Ealand, Bavesh D. Kana

**Affiliations:** 1 School of Pathology, Faculty of Health Sciences, University of the Witwatersrand, National Health Laboratory Service, Johannesburg, South Africa; Loyola University Chicago, Chicago, Illinois, USA

**Keywords:** *Mycobacterium smegmatis*, bacteriophages, mycobacteriophage, bacteriophage genetics

## Abstract

The complete genome sequences were determined for two mycobacteriophages isolated from *Mycobacterium smegmatis* mc^2^155. JayJay, a *myoviral* bacteriophage from cluster C1, encodes 234 putative genes, 32 tRNAs, and 1 tmRNA. Rinkes, a *siphoviral* bacteriophage from cluster B9, harbors a smaller genome encoding 93 putative genes.

## ANNOUNCEMENT

Bacteriophage therapy has previously been used successfully in patients with multi-drug-resistant non-tuberculous infections. This has piqued interest in identifying and characterizing new mycobacteriophages (1–4). Both mycobacteriophages in this study were isolated in Johannesburg, South Africa: JayJay from a flower bed in Berario (24 September 2022; GPS coordinates −26.1383°, 27.9526°) and Rinkes from a flowerpot in Rosebank (27 March 2023; GPS coordinates −26.1461°, 28.0432°). Respective soil samples resuspended in mycobacteriophage buffer (10 mM Tris pH 7.5, 10 mM MgSO_4_, 1 mM CaCl_2_, and 68.5 mM NaCl) were filtered through a 0.22 µm membrane, and plaques were obtained from lawns of *Mycobacterium smegmatis* mc^2^155 poured as overlays with 7H10 top-agar, incubated for 48 hours at 37°C (5). A 50 µL aliquot of the filtrate was co-incubated for 20 minutes at room temperature with 450 µL of mc^2^155 cells that had been washed twice with buffer. After serial dilution of the filtrate, a single clear plaque was picked to generate high titer stocks, which were filtered and pooled from multiple mc^2^155 lawns with near-confluent plaques flooded with buffer. These were used for negative staining transmission electron microscopy, and for DNA extraction with the Wizard Genomic DNA purification kit, according to the manufacturer’s instructions (Promega). Library preparation was performed using the NEBNext Ultra II FS kit, followed by enzymatic fragmentation (200 bp) with AMPure XP beads, end repair, ligation with Illumina-specific adapter sequences, and size selection again. Both genomes were sequenced on Illumina’s NextSeq500 platform followed by sequence trimming using Illumina Experiment Manager v1.9 and genome assembly, using default settings. The total number of paired-end reads (2 × 150 bp) was 633,907 for JayJay and 506,482 for Rinkes. The mycobacteriophage genomes were assembled and checked for quality, completeness, accuracy, and genomic termini, using Newbler v.2.9 (6) and Consed v.29.0 (7). Whole-genome nucleotide BLASTn alignments were performed at NCBI (https://blast.ncbi.nlm.nih.gov/) and PhagesDB (https://phagesdb.org/blast/). The genomes were annotated using the DNAMaster suite (v5.23.6; http://phagesdb.org/DNAMaster/) (8), Glimmer (v3.07) (9), GeneMark (v2.5p) (10), Phamerator (https://phamerator.org) (11), HHpred (https://toolkit.tuebingen.mpg.de/tools/hhpred) (12), and Aragorn V1.2.41 (13), all with default settings. The tRNAscan-SE (v2.0) (14, 15) settings were modified as follows: Sequence source: “Bacterial”; Search mode: “Infernal without HMM”; Extended Options: Check “Disable pseudo gene checking”; Check “Show primary and secondary structure components to scores”; and Genetic Code for tRNA isotype Prediction: “Universal” and a Score cut-off of 17.

**Fig 1:**
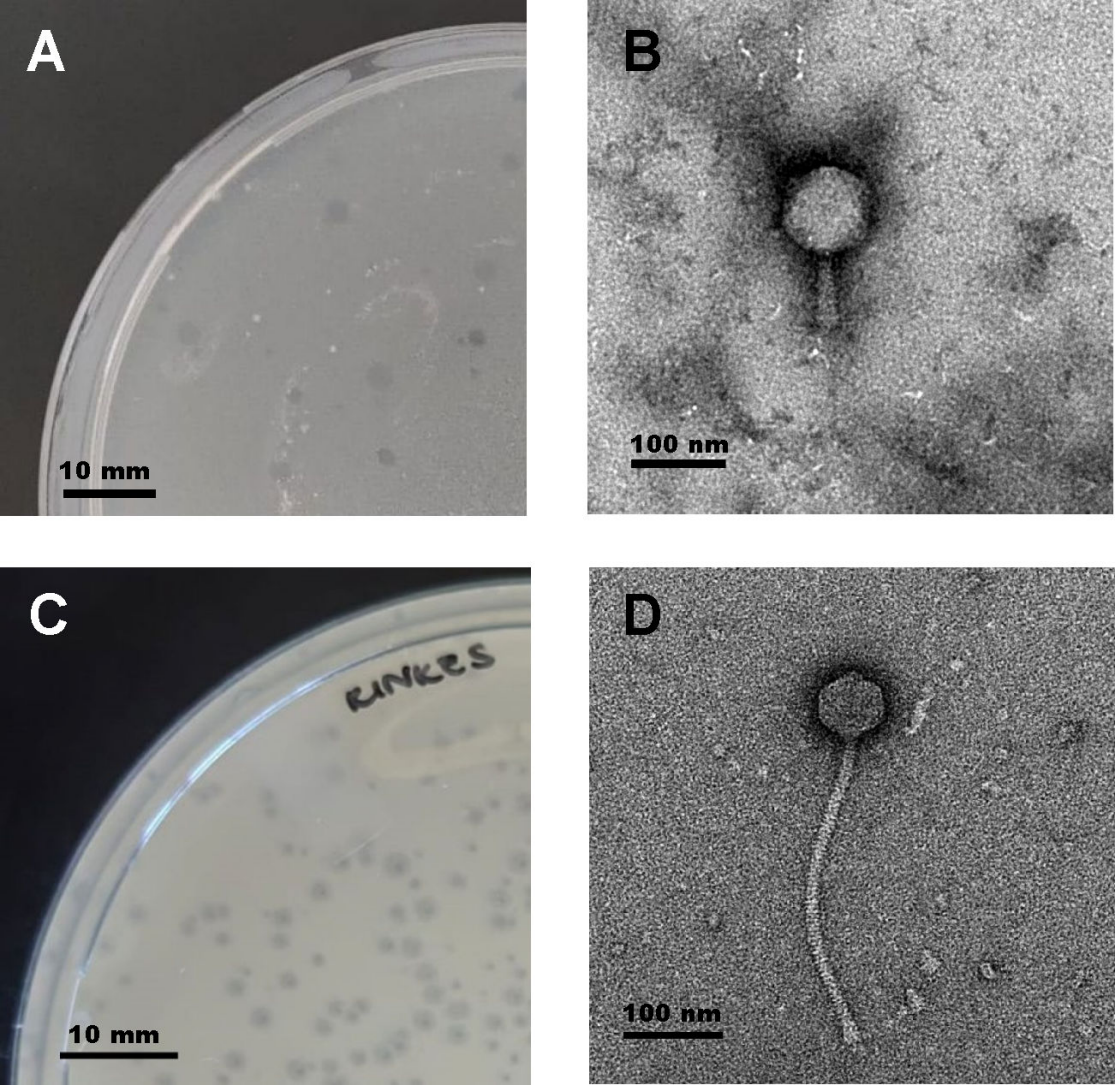
Morphological characterization of mycobacteriophages JayJay and Rinkes. (A) Plaques of mycobacteriophage JayJay on a lawn of mc2155 are clear and ~2 mm in diameter. (B) Transmission electron micrograph of a myoviral JayJay virion with an icosahedral capsule of ~100 nm diameter and a ~90 nm long contractile tail. (C) Plaques of mycobacteriophage Rinkes on a lawn of mc2155 are turbid, ~1-2 mm in diameter. (D) Transmission electron micrograph of a siphoviral Rinkes virion with an icosahedral capsule of ~64 nm diameter and a ~320 nm long contractile tail. Sizes are indicated by scale bars. Transmission electron microscopic images for the virions were derived from samples negatively stained with 2% uranyl acetate. Images were captured on a FEI T20 transmission electron microscope at 200 kV.

JayJay produced plaques of ~2 mm in diameter (Fig. 1A). The morphotype of JayJay is *myoviral* with a contractile tail (Fig. 1B). The 154,239 base pair genome (1,157-fold coverage) has a G + C content of 64.8%, is circularly permuted, and has 234 putative genes, of which 181 (77.7%) have no known function. One tmRNA and 32 tRNAs were detected. Rinkes produced clear plaques of 1–2 mm in diameter (Fig. 1C). The morphotype of Rinkes is *siphoviral* with a non-contractile tail (Fig. 1D). The 70,563 base pair genome (2,144-fold coverage) has a G + C content of 69.6%, is circularly permuted, and has a total of 93 predicted genes, 46 (49.5%) of which have no known function.

## Data Availability

Genome data for phage JayJay are available at GenBank under accession number PQ449569.1, and at the Sequence Read Archive under SRR28773258. Genome data for phage Rinkes are available at GenBank under accession number PQ449568.1, and at the Sequence Read Archive under SRR30691026.
